# Association between Common Genetic Variants in the Opioid Pathway and Smoking Behaviors in Chinese Men

**DOI:** 10.1186/1744-9081-10-2

**Published:** 2014-01-21

**Authors:** Juan Fang, Xiaohong Wang, Bei He

**Affiliations:** 1 Department of Respiratory Medicine, Peking University Third Hospital, No. 49 Hua Yuan Bei Road, Haidian District, Beijing 100191, China

**Keywords:** Smoking, Polymorphisms, Mu-opioid receptor, *ARRB2*, *HINT1*

## Abstract

**Background:**

There is biological evidence that the brain opioidergic system plays a critical role in the addictive properties of nicotine. The purpose of the present study was to examine the associations of single nucleotide polymorphisms (SNPs) in the genes encoding mu-opioid receptor (MOR) and the MOR-interacting proteins (including *OPRM1*, *ARRB2*, and *HINT1*) with smoking behaviors in Chinese men.

**Methods:**

A total of 284 subjects (including current and ex-smokers) were recruited. Special questionnaires were used to assess smoking behaviors including age of smoking initiation, daily cigarette consumption, and Fagerström test for nicotine dependence (FTND) score. Participant samples were genotyped for six SNPs in the opioid pathway genes: rs1799971 in *OPRM1*, rs1045280, rs2036657 and rs3786047 in *ARRB2*, rs3852209 and rs2278060 in *HINT1*. Linear and logistic regression models were used to determine single-locus and haplotype-based association analyses.

**Results:**

There was no significant association between any of SNPs analyzed and smoking behaviors. Logistic regression analyses under dominant, recessive, and additive models showed no significant associations of the six SNPs with smoking status (current vs. ex-smokers). After adjustment for age at enrollment and smoking initiation age, *HINT1* rs3852209 was significantly associated with smoking status with an OR of 0.54 (95% CI, 0.31-0.95; *P* = 0.03) under dominant inheritance model. No haplotypes in *ARRB2* or *HINT1* were related to smoking status.

**Conclusions:**

The present study indicates no significant association between common genetic variations in MOR and MOR-interacting proteins and smoking behaviors in Chinese men, and gives suggestive evidence that *HINT1* rs3852209 may be related to smoking status. The findings require confirmation from further studies in additional larger samples.

## Introduction

Tobacco use remains the leading preventable cause of death in the world. According to the World Health Organization (WHO), tobacco use is responsible for the death of approximately six million people and causes hundreds of billions of dollars of economic loss worldwide each year [1]. In 2010, the Global Adult Tobacco Survey (GATS) China conducted by the Chinese Center for Disease Control and Prevention reported that 28.1% of adults in China were current smokers, and the prevalence of smoking in men was significantly higher than that in women (52.9% vs. 2.4%) [1]. There was an estimation of 301 million current smokers in China, making this country the largest consumer of tobacco in the world [2]. Cigarette smoking increases the risk of numerous health problems and causes a variety of tobacco-related diseases, including primarily cancers, diabetes, cardiovascular diseases, and chronic lung diseases. Up to half of the world’s 1 billion smokers will eventually die from a tobacco-related disease [1].

Cessation reverses most adverse effects of smoking. The majority of smokers are highly motivated to quit, but confronted with high relapse rate after stopping smoking [3]. The same issue is confronting the patients with tobacco-related diseases. Although some smoking patients quit because of diseases, there is still a considerable proportion of them who persist smoking even after being informed that tobacco smoking is an independent risk factor for their diseases. As several surveys revealed, the percentage of current smokers accounting for the patients with tobacco-related diseases was compared to that of general population [4-6]. Despite great progress in pharmacological treatment of nicotine dependence, the efficacy of available treatments is limited, and only 15–30% of smokers permanently abstain from smoking [3].

Tobacco smoking is believed to be a complex and multifactorial behavior with both genetic and environmental determinants. Recent researches strongly suggested that smokers varied in their underlying genetic susceptibility to smoking addiction, and have found significant genetic influences on several aspects of smoking behaviors, including smoking initiation, smoking maintenance and cessation success [7,8]. Variations in several genes have been suggested to contribute to smoking behaviors, and these researches have been focused on two broad classes of candidate genes: 1) genes that may influence the response to nicotine (e.g. nicotine metabolism and nicotinic receptors) and 2) genes that may predispose to addictive behavior because of their effects on key neurotransmitter pathways (e.g. dopamine and serotonin) [9,10].

Although multiple neurobiological mechanisms have been implicated in mediating the reward and reinforcing effects of drug abuse including nicotine, for the past decade increasing studies pointed to the endogenous opioid system, especially the mu-opioid receptor (MOR) [11,12]. Genetic and pharmacological studies indicated that mu-opioid receptors were mainly involved in nicotine reward [10,13-16]. These findings have led us to consider the potential role of variations in genes encoding proteins regulating the function of the MOR. Several proteins were manifested to interact with the MOR in in-vitro models and had some opioid-related phenotypes demonstrated in mouse knock-out models [17]. However, it remains uncertain whether and to what extent common genetic variants in the genes of MOR interacting proteins affect smoking behaviors on account of limited population data.

Considering higher prevalence of smoking in Chinese men compared with women, we conducted a cross-sectional study in Chinese male smokers to explore the associations between smoking behaviors and genetic variants in the MOR and MOR-interacting protein genes, including *OPRM1* gene encoding mu-opioid receptor and two genes encoding mu opioid-receptor-interacting proteins, namely *ARRB2* and *HINT1*.

## Materials and methods

### Subjects

Participants were recruited at In-patient Departments of a general hospital (Peking University Third Hospital) from February 2011 to February 2012, including Respiratory Medicine, Cardiology and Endocrinology Departments. A total of 284 Chinese males suffering from tobacco-related diseases were included in this study. All participants reported themselves as Han nationality and Beijing local residents. Inclusion criteria were: smokers (continuous or cumulative smoking six months or more in lifetime, including current smokers and ex-smokers), aged 18–80 years old, with one or more of tobacco-related diseases (including coronary heart disease, atherosclerosis, cerebrovascular disease, chronic obstructive pulmonary disease, bronchial asthma, and diabetes mellitus). Exclusion criteria were: patients with drug or alcohol addiction, history of psychiatric illnesses other than nicotine dependence, or serious clinical conditions and inability to communicate. In view of remarkable gender difference in cigarette smoking in China, only male subjects were involved in the present study to eliminate the possible gender effect.

In accordance with smoking status at enrollment, participants were classified into current smokers and ex-smokers, which were defined as follows: 1) current smokers were subjects who either were currently smoking at enrollment or had abstained from smoking for less than one year before interview; 2) ex-smokers were those who remained continuously abstinent from cigarettes for at least one year before the interview. Considering the effect of disease history on smoking behaviors, the participants were classified into three groups based on their main tobacco-related diseases diagnosed at admission: cardiovascular and cerebrovascular disease, respiratory disease, and diabetes mellitus.

### Questionnaire

All individuals provided informed consent for participation in this study and completed questionnaires assessing demographics and smoking behaviors (including age of smoking initiation, daily cigarette consumption, smoking years, and Fagerström test for nicotine dependence (FTND) score at enrollment) [18]. The study protocol was approved by the ethics committee of Peking University Third Hospital.

### Genotyping

Peripheral blood was drawn during the enrollment visit, transported to a central laboratory and stored at -80°C for further analysis. DNA was extracted from blood leukocytes using a commercial DNA extraction kit (RelaxGene Blood DNA System, TIANGEN BIOTECH (BEIJING) CO., LTD.).

Candidate SNPs were selected with regard to minor allele frequency and location in the genes of *HINT1* and *ARRB2* from the 5' to 3' regions [19], and practical considerations regarding assay design and resource limitations. Variants included: rs1799971, an Asn40Asp (A118G) polymorphism in exon 1 of the *OPRM1* gene located on chromosome 6; rs1045280, rs2036657 and rs3786047 in *ARRB2*, a 11-kb gene located on chromosome 17; rs3852209 and rs2278060 in *HINT1*, a 6-kb gene located on chromosome 5. Using Haploview analysis, high linkage disequilibriums for SNPs in *HINT1* (rs3852209 & rs 2278060: D' = 1.0, r^2^ = 0.921) and *ARRB2* (rs 1045280 & rs 2036657: D' = 0.987, r^2^ = 0.973; rs 1045280 & rs 3786047: D' = 1.0, r^2^ = 0.987; rs 2036557 & rs 3786047: D' = 0.986, r^2^ = 0.96) were observed.

Gnotypes were determined by iPLEX/MALDI-TOF mass spectrometry (Sequenom, Inc.), applying extensive quality control measures. The capture and extend primers of SNPs for genotyping were designed with reference to NCBI genome build 37.1. The genotyping results were validated by repeating 5% of the total sample; no genotyping errors were detected. The genotyping call rate for all subjects was 99.6%-100%.

### Statistical analysis

The SPSS 19.0 statistical package was used in the following data analyses. Chi-square tests and t-tests were used to test differences of baseline variables by smoking status. The distribution of genotype was compared with predictable value of the Hardy-Weinberg heredity equilibrium by the Pearson χ2 test. The associations of SNPs with smoking status were analyzed by using logistic regression models adjusted for age at enrollment and smoking initiation age, which were expressed as odds ratio (OR) and 95% confidence interval (CI). To determine the associations between the genotypes and smoking initiation age, daily cigarette consumption and FTND score respectively, general linear models were used to compare the differences of continuous variables among different genotype groups. Age as a covariate was included in linear models. Linkage disequilibrium was examined by using Haploview version 4. We constructed haplotypes using PHASE version 2.0 and examined the associations of haplotypes with smoking status by logistic regression models. Rare haplotypes (<1%) were omitted from the analyses. For all analyses, difference with a two-sided P < 0.05 was considered statistically significant.

## Results

### Characteristics of the subjects

Of all 284 smokers in the study, 146 (51.4%) were ex-smokers who successfully quit smoking and remained abstinent for at least one year. The demographic and clinical characteristics and smoking behaviors were shown by smoking status in Table 1 (age: 62.6 ± 13.4 years [mean ± SD], education: education of 64.8% participants were more than 9 years, smoking status: 138 current smokers and 146 ex-smokers). Compared with ex-smokers, current smokers were significantly younger (P < 0.05) at enrollment, and smoking initiation age was earlier (P < 0.05) (Table 1). For ex-smokers, the average age of quitting was 59.9 years old (SD, 9.8), and the average length of time since quitting was 8.6 years (SD, 8.4). 115 (83.3%) of current smokers tried to quit repeatedly and then relapsed even after a short period of abstinence.

**Table 1:** Characteristics, smoking behaviors and distribution of genotypes by smoking status

**Characteristics**	**Current smokers (n = 138)**	**Ex-smokers (n = 146)**	**P value**^**c**^
**Age (years)**^**a**^	56.4 ± 14.8	68.5 ± 8.3	<0.05
**Education**^**b**^			
< 9 years	49 (35.5)	51 (34.9)	0.92
≥ 9 years	89 (64.5)	95 (65.1)	
**Disease history**^**b**^			
Cardiovascular and cerebrovascular diseases	81 (58.7)	91 (62.3)	0.15
Respiratory diseases	38 (27.5)	45 (30.8)	
Diabetes mellitus	19 (13.8)	10 (6.8)	
**Smoking behaviors**^**a**^			
Age of smoking initiation (years)	23.6 ± 8.0	28.3 ± 10.8	<0.05
Daily cigarette consumption (per day)	22.3 ± 11.9	19.9 ± 9.9	0.06
Smoking years (years)	32.9 ± 14.3	31.7 ± 12.3	0.46
**Genotype**^**b**^			
**rs1799971**^**d**^			
AA	64 (46.7)	72 (49.3)	0.54
AG	62 (45.3)	58 (39.7)	
GG	11 (8.0)	16 (11.0)	
**rs1045280**			
CC	2 (1.4)	2 (1.4)	0.99
CT	39 (28.3)	41 (28.1)	
TT	97 (70.3)	103 (70.5)	
**rs2036657**			
AA	98 (71.0)	103 (70.5)	0.93
AG	38 (27.5)	40 (27.4)	
GG	2 (1.4)	3 (2.1)	
**rs3786047**^**d**^			
AA	1 (0.7)	2 (1.4)	0.87
AG	38 (27.5)	41 (28.1)	
GG	98 (71.0)	103 (70.5)	
**rs3852209**			
CC	79 (57.2)	99 (67.8)	0.11
CT	53 (38.4)	39 (26.7)	
TT	6 (4.3)	8 (5.5)	
**rs2278060**			
AA	77 (55.8)	94 (64.4)	0.28
AC	54 (39.1)	44 (30.1)	
CC	7 (5.1)	8 (5.5)	

^a^Values are means ± SD.

^b^Values are n (%). ^c^P value from chi-square and t tests.

^d^Genetic material for genotyping was available for 283 of the subjects.

The proportions of cardiovascular & cerebrovascular diseases and respiratory diseases in ex-smokers were higher than those in current smokers, while the proportions of diabetes mellitus in ex-smokers and current smokers were opposite. But the results of Pearson chi-square test indicated that the difference in disease history between current smokers and ex-smokers did not reach the significant level (P = 0.15). Multiple comparisons of various disease histories between ex-smokers and current smokers showed there were no significant differences (cardiovascular & cerebrovascular disease vs. respiratory disease: P = 0.84; cardiovascular & cerebrovascular disease vs. diabetes mellitus: P = 0.07; respiratory disease vs. diabetes mellitus: P = 0.07).

Table 2 listed the characteristics of the selected SNPs. The minor allele frequencies overall were in concordance with HapMap reference data (Table 2). The genotype distributions of SNPs in current smokers and ex-smokers shown in Table 1 fulfilled the expectations of the Hardy–Weinberg equilibrium.

**Table 2:** Genotype quality control indicators

**Gene**	**Genotype identifier**	**Chromosome position**	**Function**	**Minor allele frequency (minor allele)**		**p-value#**
				***HapMap-CHB***	***Study population***	
*ARRB2*	rs3786047	4615098	intron	0.161 (A)	0.153 (A)	0.34
*ARRB2*	rs1045280	4622638	synonymous	0.167 (C)	0.155 (C)	0.30
*ARRB2*	rs2036657	4625159	3' near gene	0.117 (G)	0.155 (G)	0.59
*HINT1*	rs3852209	130496015	intron	0.225 (T)	0.211 (T)	0.73
*HINT1*	rs2278060	130500751	intron	0.250 (C)	0.225 (C)	0.94
*OPRM1*	rs1799971	154360797	missense	0.361 (G)	0.307 (G)	1

^#^Calculated by using *χ*2 test (deviation from Hardy–Weinberg conditions).

### Polymorphisms and smoking behaviors

General linear models revealed no significant differences of age of smoking initiation or daily cigarette consumption in various genotypes of each SNP. The degree of nicotine dependence of current smokers was ascertained by the FTND score, and the average FTND score was 3.7 (SD, 2.6). There were no significant associations between SNPs and FTND scores in current smokers (Table 3).

**Table 3:** Associations of genotypes with smoking initiation age (in years), daily cigarette consumption (in cigarettes per day) and FTND score as continuous traits

**Genotype**	**Smoking initiation age**^**a**^	**Daily cigarette consumption**^**a**^	**FTND score**^**ac**^
**rs1799971**			
AA	25.8 ± 9.3	20.6 ± 10.3	4.1 ± 2.6
AG	25.9 ± 10.1	22.1 ± 12.0	3.5 ± 2.6
GG	27.6 ± 11.0	18.9 ± 8.9	2.7 ± 2.2
β Coefficient (se)	0.05 (1.00)	0.48 (0.84)	–0.63 (0.35)
P value^b^	0.96	0.56	0.07
**rs3786047**			
AA	26.0 ± 11.8	21.7 ± 17.6	8.0 ± 0.0
AG	24.9 ± 10.2	21.1 ± 9.8	3.9 ± 2.7
GG	26.5 ± 9.7	21.0 ± 11.4	3.6 ± 2.5
β Coefficient (se)	0.05 (1.36)	–1.11 (1.13)	0.58 (0.47)
P value^b^	0.97	0.33	0.22
**rs1045280**			
CC	23.5 ± 10.8	21.3 ± 14.4	5.0 ± 4.2
CT	24.8 ± 10.2	21.1 ± 9.8	3.9 ± 2.6
TT	26.6 ± 9.7	21.1 ± 11.4	3.6 ± 2.5
β Coefficient (se)	–0.14 (1.33)	–1.16 (1.11)	0.45 (0.45)
P value^b^	0.92	0.29	0.32
**rs2036657**			
AA	26.6 ± 9.6	21.2 ± 11.4	3.7 ± 2.6
AG	24.9 ± 10.2	20.8 ± 9.7	3.8 ± 2.6
GG	21.6 ± 10.3	21.0 ± 12.4	5.0 ± 4.2
β Coefficient (se)	–0.39 (1.31)	–1.35 (1.09)	0.32 (0.45)
P value^b^	0.76	0.22	0.49
**rs3852209**			
CC	26.4 ± 10.5	21.2 ± 11.5	3.5 ± 2.5
CT	25.5 ± 8.7	21.2 ± 10.1	4.2 ± 2.6
TT	24.9 ± 7.9	18.8 ± 9.0	2.5 ± 2.6
β Coefficient (se)	–0.60 (1.11)	–0.69 (0.93)	0.19 (0.38)
P value^b^	0.59	0.46	0.62
**rs2278060**			
AA	26.3 ± 10.6	21.4 ± 11.7	3.5 ± 2.5
AC	25.9 ± 8.6	20.9 ± 9.9	4.2 ± 2.6
CC	23.9 ± 8.4	18.9 ± 8.7	2.4 ± 2.4
β Coefficient (se)	–0.86 (1.09)	–0.70 (0.92)	0.12 (0.37)
P value^b^	0.43	0.44	0.75

^a^Values are means ± SD.

^b^Linear regression models were adjusted for age.

^c^Current smokers.

### Polymorphisms and smoking status

To estimate the influence of genetic variation on smoking status, the genotype frequencies of each SNP were compared by Pearson χ^2^ test between current smokers and ex-smokers, and no statistically significant differences were found (Table 1).

Logistic regression models were performed to calculate ORs and 95% CIs for SNPs with the probability of being ex-smokers. For each SNP, we tested for allelic associations with smoking status under dominant, recessive, and additive models. All SNPs analyzed had no associations with smoking status (Table 4). After adjustment for age at enrollment and age of smoking initiation, logistic regression models showed that rs3852209 in *HINT1* gene was associated with smoking status under the dominant model and the OR was 0.54 (95% CI: 0.31-0.95, *P* < *0.05*), indicating the individuals with CC genotype had higher probabilities of smoking cessation compared with those with T allele (i.e. CT and TT genotypes). Besides, age was significantly associated with smoking cessation, the probability of successful quitting increased with age (OR = 1.09, 95% CI: 1.06-1.11, *P* < *0.05*).

**Table 4:** Associations of SNPs with smoking status (ex-smokers vs. current smokers)

**Model**	**Crude OR (95% CI)**^**a**^	**P value**	**Adjusted OR (95% CI)**^**b**^	**P value**
**rs1799971**				
Additive	1.00 (0.70–1.43)	0.96	0.99 (0.66–1.48)	0.97
AA vs. AG vs. GG				
Dominant	0.90 (0.56–1.43)	0.66	0.87 (0.51–1.48)	0.62
AA vs. AG + GG				
Recessive	1.41 (0.63–3.15)	0.40	1.40 (0.57–3.42)	0.46
AA + AG vs. GG				
**rs3786047**				
Additive	1.07 (0.66–1.74)	0.77	1.25 (0.71–2.19)	0.42
GG vs. GA vs. AA				
Dominant	1.04 (0.62–1.75)	0.85	1.23 (0.68–2.22)	0.47
GG vs. GA + AA				
Recessive	1.88 (0.16–21.07)	0.60	2.49 (0.14–43.07)	0.52
GG + GA vs. AA				
**rs1045280**				
Additive	0.98 (0.61–1.58)	0.95	1.21 (0.70–2.09)	0.48
TT vs. TC vs. CC				
Dominant	0.98 (0.59–1.64)	0.96	1.21 (0.67–2.19)	0.50
TT vs. TC + CC				
Recessive	0.94 (0.13–6.79)	0.95	1.48 (0.14–15.76)	0.74
TT + TC vs. CC				
**rs2036657**				
Additive	1.04 (0.65–1.66)	0.85	1.26 (0.73–2.16)	0.40
AA vs. AG vs. GG				
Dominant	1.02 (0.61-1.70)	0.93	1.25 (0.69-2.26)	0.45
AA vs. AG + GG				
Recessive	1.42 (0.23–8.66)	0.70	1.88 (0.21–16.24)	0.56
AA + AG vs. GG				
**rs3852209**				
Additive	0.75 (0.50–1.13)	0.17	0.72 (0.45–1.13)	0.15
CC vs. CT vs. TT				
Dominant	0.63 (0.39–1.03)	0.06	**0.54 (0.31–0.95)**	**0.03**
CC vs. CT + TT				
Recessive	1.27 (0.43–3.77)	0.66	1.75 (0.52–5.90)	0.36
CC + CT vs. TT				
**rs2278060**				
Additive	0.79 (0.53–1.17)	0.24	0.76 (0.49–1.20)	0.24
AA vs. AC vs. CC				
Dominant	0.69 (0.43–1.12)	0.14	0.60 (0.35–1.03)	0.06
AA vs. AC + CC				
Recessive	1.08 (0.38–3.07)	0.87	1.56 (0.48–5.03)	0.45
AA + AC vs. CC				

^a^Crude ORs were calculated with 2 × 2 cross-tabulation.

^b^Adjusted ORs were obtained from binary logistic regression.

OR, odds ratio; CI, confidence interval.

### Haplotype analysis

For the *ARRB2* SNPs rs 3786047, rs 1045280 and rs 2036657, respectively, the common haplotypes were A-C-G (15.1%) and G-T-A (84.3%), with the remainder totaling 0.5%. For the *HINT1* SNPs rs 3852209 and rs 2278060, respectively, the two common haplotypes were C-A (77.5%) and T-C (21.1%), and the other C-C was 1.4%. The frequencies of haplotypes in *ARRB2* and *HINT1* had no significant differences between current smokers and ex-smokers (*P* > 0.05). No haplotypes showed significant associations with smoking status. After adjustment for age at enrollment, smoking initiation age and disease history, no significant associations were observed between the haplotypes and smoking status (Table 5).

**Table 5:** Associations between haplotypes and smoking status

**Gene**	**Haplotype**	**Crude OR (95% CI)**	**P value**	**Adjusted OR (95% CI)**	**P value**
***ARRB2***					
**rs3786047–rs1045280–rs2036657**					
	G-T-A	1		1	
	A-C-G	1.04 (0.65–1.64)	0.86	1.26 (0.74–2.15)	0.38
***HINT1***					
**rs3852209–rs2278060**					
	C-A	1		1	
	C-C	1.31 (0.87–1.97)	0.18	1.35 (0.85–2.15)	0.19
	T-C	1.97 (0.45–8.61)	0.36	2.04 (0.40–10.24)	0.38

## Discussion

The present study focused on the potential association of SNPs in genes encoding MOR (*OPRM1*) and MOR-interacting proteins (*ARRB2* and *HINT1*) with smoking behaviors. The results generated from Chinese men with tobacco-related diseases suggested that *HINT1* rs3852209 was significantly associated with smoking status (current smokers vs. ex-smokers). However, it did not provide evidence for the effect of variations in *OPRM1*, *ARRB2* and *HINT1* genes on smoking behaviors including age of smoking initiation, daily cigarette consumption, and FTND score. In addition, the logistic regression analyses revealed that smokers were increasingly probable to achieve smoking cessation with age independent of the other covariates, which was consistent with the results described in previous trials and survey studies [20,21].

Recently, we have demonstrated that there was a considerable percentage of smoking patients with tobacco-related diseases did not successfully quit smoking, and were still current smokers. Most of these patients were willing to quit, especially after having recognized tobacco smoking as an independent risk factor for their diseases. But unfortunately, they were confronted with as high rate of relapse as general population [22]. From the above, it can be seen why an ex-smoker is defined as one who has quit smoking for at least one year. This assertion is supported by the previous evidence that self-reported cessation for at least one year had the highest likelihood of continuous abstinence of smoking [23]. In the present study, 83.3% of current smokers attempted to quit and used to be abstinent for a short time, but finally relapsed. Although the average age of current smokers was lower than that of ex-smokers, both of current smokers and ex-smokers had the same number of smoking years at enrollment. Thus, it was indicated that current smokers initiated smoking earlier than ex-smokers. Overall, the present study revealed the differences of smoking behaviors between current smokers and ex-smokers, and suggested that current smokers might have more difficulties in quitting.

Although the mesolimbic dopamine hypothesis is the most influential theory of nicotine reward and reinforcement, recent developments in the neurobiology of nicotine dependence have identified several other neurotransmitter systems that may contribute to the addictive properties of nicotine as well. In this regard, the brain opioidergic system has been of interest. Nicotine stimulates the release of endogenous peptide β-endorphin which has the high affinity for the mu-opioid receptor [24]. The activated mu-opioid receptors suppress the release of neurotransmitter GABA [25]. Thus, the decrease of GABA level causes the disinhibition of VTA dopamine neurons, which leads to the increase of dopamine release from nucleus accumbens [26]. Presumably, it is the association between the opioidergic system and the mesolimbic dopamine system that appeals to explore the role of mu-opioid receptors in smoking behaviors. The previous studies using inbred and knockout mice strongly indicated that the mu-opioid receptor mediated both the positive and negative reinforcing effects of nicotine. And it was found that nicotine induced reward effect and dependence was substantially attenuated in mu-opioid receptor knock-out mice [10,13]. All these suggest that mu-opioid receptor, encoded by the gene of *OPRM1*, is one of the factors contributing to tobacco addiction, and thus the *OPRM1* becomes an attractive candidate gene for smoking behaviors in humans. The Asn40Asp (A118G) polymorphism (rs1799971), located in exon I of *OPRM1* gene, induces functional change of the mu-opioid receptor and becomes one of the most studied polymorphisms [27]. This mis-sense SNP rs1799971 causes the substitution of an aspartate (Asp) for an asparagine (Asn) at position 40 in the amino-acid sequence. The Asp40 (G) variant, related to the reduced expression of *OPRM1,* was found to increase the binding affinity of β-endorphin for the mu-opioid receptor by three-fold compared with the wild-type Asn40 (A) *OPRM1*[13], nevertheless this finding subsequently failed to be replicated [28,29]. In rodent studies, there was evidence that *OPRM1* G allele might play a role in susceptibility to nicotine addiction [11]. In the clinical trial, Lerman and colleagues found that Asn40Asp (A118G) mis-sense SNP in *OPRM1* might predict the treatment responses to NRT (nicotine replacement therapy). Specifically, the smokers with one or more copies of G allele (Asp40 variant) were more likely to quit after a course of NRT than individuals with two copies of A allele [16]. Their results might be explained by the fact that smokers with G allele could gain more reward from nicotine provided by NRT and then derived greater benefit from NRT. However, the present study did not provide strong support for the role of the *OPRM1* polymorphism (rs1799971) in smoking behaviors. To be specific, there were no statistical associations identified between the *OPRM1* Asn40Asp (A118G) polymorphism and smoking status as well as other smoking behaviors (including age at smoking initiation, daily cigarette consumption and nicotine dependence) in Chinese males.

Furthermore, the mu-opioid receptor directly interacts with multiple proteins called MOR interacting proteins (MORIPs), and thus MORIPs gene polymorphisms are considered to influence on the individual susceptibility to drug dependence. Of the interacting proteins, β-arrestin 2 and mPKCI-1 have demonstrable opioid-related phenotypes in mouse knock-out model and other in-vitro models [17]. By binding to phosphorylated MOR, β-arrestin 2 has been found to be an important regulator of signal transduction mediated by opioid receptors through promotion of receptor desensitization and internalization and plays a role in opioid reward [17,30,31]. *ARRB2* is known as the gene encoding β-arrestin 2. In *ARRB2* null mutant animals, MOR did not undergo desensitization and then the increased and prolonged antinociceptive effects were perceived after morphine challenge [32]. In contrast to β-arrestin 2, mPKCI-1 (known as histidine triad nucleotide binding protein 1 in humans) encoded by the gene *HINT1* decreases MOR phosphorylation and desensitization. It has been shown that mPKCI-1 knock-out mice could develop tolerance to analgesic effect of morphine much more quickly than wild-type mice [33]. In the present study, the results indicated a significant association between *HINT1* variant rs3852209 and smoking status. Compared with CC genotype, the subjects with *HINT1* rs3852209 T allele were less possible to achieve smoking cessation. Although it is unknown how *HINT1* rs3852209 T allele affects *HINT1* gene expression, presumably T allele leads to the deficiency of mPKCI-1 expression, which is incapable to inhibit MOR desensitization. As a result, the individual with T allele develops tolerance to nicotine and is susceptible to nicotine dependence and then less likely to quit smoking. The haplotype analyses did not provide evidence for the relationship between *ARRB2* or *HINT1* and smoking status, which is consistent with the results from the previous clinical NRT trial [19].

After all, this is the primary study exploring the role of genetic variations of MOR-interacting proteins in smoking behaviors. Several limitations in our study should be considered. First, one major limitation of this study was the limited sample size with the power about 20% (OR: 1.2 - 1.3) calculated by Quanto, which was not sufficient to determine the associations between genetic variations and smoking behaviors and might lead to false negative results which meant missed potential positive associations. Given the minor genetic effects, the results of our study might be caused by the limited sample size. Further studies with sufficient statistical power are required to verify the present results. Second, the mismatch of age at enrollment between current smokers and ex-smokers might influence the results to a certain extent, though the adjustments were adopted. Third, the study focused on the subjects with tobacco-related diseases enrolled from a general hospital. Considering the variety of diseases and differences in severity and course of the diseases, it was difficult to precisely determine the effect of tobacco-related disease on smoking behavior. At last, smoking history was accessed by the participants' self-report without biochemical confirmation for current smoking status, which might possibly cause misclassification.

Despite these limitations, the present study preliminarily investigated the role of genetic variations of the opioid pathway in smoking behaviors of Chinese men. Our results revealed no significant association between common genetic variations in genes encoding MOR and MOR-interacting proteins and smoking behaviors of Chinese men, and presented suggestive evidence for the association between the MOR-interacting protein encoding gene and smoking status (current smokers vs. ex-smokers). Finally, further prospective studies need to be replicated in order to confirm the findings of the present study.

## Competing interests

We declare that all the authors have no competing interests.

## Authors’ contributions

JF have made contributions to conception and design, acquisition of data, genotyping, analysis and interpretation of data, and draft the manuscript. XW participated in the design of the study and acquisition of data. BH conceived of the study, and participated in its design and coordination and helped to revise the manuscript critically for important intellectual content. All authors read and approved the final manuscript.
